# Proenkephalin A and bioactive adrenomedullin are useful for risk prognostication in cardiac surgery

**DOI:** 10.3389/fcvm.2022.1017867

**Published:** 2023-01-23

**Authors:** Aileen Hill, Deborah Bergmann, Janin Schulte, Rashad Zayat, Gernot Marx, Tim-Philipp Simon, Jana Mossanen, Anne Brücken, Christian Stoppe

**Affiliations:** ^1^Department of Intensive Care and Intermediate Care, Medical Faculty RWTH Aachen, Aachen, Germany; ^2^Department of Anesthesiology, Medical Faculty RWTH Aachen, Aachen, Germany; ^3^SphingoTec GmbH, Hennigsdorf, Germany; ^4^Department of Cardiothoracic Surgery, Medical Faculty RWTH Aachen, Aachen, Germany; ^5^Department of Anesthesiology, Intensive Care, Emergency and Pain Medicine, University Hospital Würzburg, Würzburg, Germany

**Keywords:** cardiac surgery, critical care, shock, prognosis, biomarkers, proenkephalin A, adrenomedullin, acute kidney injury

## Abstract

**Introduction:**

Various clinical scores have been developed to predict organ dysfunction and mortality in patients undergoing cardiac surgery, but outcome prediction may be inaccurate for some patient groups. Proenkephalin A (penKid) and bioactive adrenomedullin (bio-ADM) have emerged as promising biomarkers correlating with shock and organ dysfunction. This imposes the question of whether they can be used as prognostic biomarkers for risk stratification in the perioperative setting of cardiac surgery.

**Methods:**

Patients undergoing cardiac surgery were prospectively enrolled in this observational study. PenKid and bio-ADM plasma levels, as well as markers evaluating inflammation and organ dysfunction, were measured at five perioperative time points from before the induction of anesthesia to up to 48 h postoperatively. Clinical data regarding organ dysfunction and patient outcomes were recorded during the intensive care unit (ICU)-stay with a special focus on acute kidney injury (AKI).

**Results:**

In 136 patients undergoing cardiac surgery, the bio-ADM levels increased and the penKid levels decreased significantly over time. PenKid was associated with chronic kidney disease (CKD), the incidence of AKI, and renal replacement therapy (RRT). Bio-ADM was associated with lactate and the need for vasopressors. PenKid was useful to predict an ICU-length of stay (LOS)>1 day and added prognostic value to the European System for Cardiac Operative Risk Evaluation Score (EuroSCORE) II when measured after the end of cardiopulmonary bypass and 24 h after cardiac surgery. For bio-ADM, the same was true when measured 24 h after surgery. PenKid also added prognostic value to the EuroSCORE II for the combined outcome “ICU length of stay >1 day and in-hospital mortality.”

**Conclusion:**

The combination of preoperative EuroSCORE II and intraoperative measurement of penKid may be more useful to predict a prolonged ICU LOS and increased mortality than EuroSCORE II alone. Bio-ADM correlates with markers of shock. More research is encouraged for early risk stratification and validation of penKid and bio-ADM as a tool involved in clinical decisions, which may enable the early initiation of organ protective strategies.

## 1. Introduction

Patients undergoing cardiac surgery are exposed to hemodynamic instability, oxidative stress, systemic inflammation, drugs, and toxins with consecutive endothelial dysfunction and vasodilatation. Consequences are capillary leakage with a decrease in intravascular volume, insufficient cardiac preload, and general edema, promoting hypotension in a vicious circle. If it is left untreated, shock and organ damage such as acute kidney injury (AKI) are possible consequences (1–3). Organ dysfunctions often necessitate a prolonged intensive care unit (ICU) stay and strongly influence short- and long-term outcomes, such as length of stay (LOS), quality of life, and mortality in patients undergoing cardiac surgery (1–4).

These patients may benefit from early risk stratification and detection of complications such as shock and organ dysfunction. Nevertheless, risk stratification remains complicated. Several risk models were developed to predict procedural mortality in patients undergoing cardiac surgery. The European System for Cardiac Operative Risk Evaluation (EuroSCORE) is a system for the prediction of early mortality in patients undergoing cardiac surgery (5). The updated EuroSCORE (EuroSCORE II) (6) and the Society of Thoracic Surgeons (STS) risk model (7, 8) are currently widely accepted (9, 10). However, these models may not perform accurately in all patients regarding the heterogeneity of this patient population and procedures. According to the European Association for Cardio-Thoracic Surgery (EACTS), particularly in high-risk patients, the current risk models have been shown to be poorly calibrated, indicating a need for quality improvement in risk prediction (11).

The addition of biomarkers to established clinical risk models may improve the predictive value (12) and can help identify high-risk patients with poor clinical outcomes, which may benefit from specific treatments (13). Adequately validated biomarkers in clinical trials could reduce heterogeneity between the patients and thus reduce the sample size, improve the statistical power, and save promising therapies from false-negative results in clinical trials (13). However, the biomarkers currently used in the clinical routine are often troublesome, as they react too late, are influenced by various factors, and thus may detect a diverse patient population (13–15). Previous attempts to add biomarker panels to a risk model in cardiac surgery did not yield satisfactory results or have not been successfully integrated into clinical routines so far (16, 17). Therefore, novel and more specific biomarkers correlating closely with the clinical course are urgently needed for a more effective treatment of patients at risk for severe postoperative complications.

Proenkephalin A (penKid) has emerged as a promising and reliable biomarker for the detection of AKI (18). As an endogenous opioid, it is produced in a variety of non-neuronal tissues (19) and may influence various biochemical pathways, such as cardiovascular function and inflammation (20). PenKid correlates with high serum creatinine levels and low glomerular filtration rate (21). Elevated penKid levels predict AKI and the need for renal replacement therapy (RRT) in patients undergoing cardiac surgery (22, 23), after acute myocardial infarction (24), in the ICU (25), in the general population (26), as well as in patients with sepsis (20). PenKid plasma levels increase with the severity of AKI and correlate with short-term mortality in patients with sepsis (27). In ambulatory patients with heart failure and acute myocardial infarction, elevated penKid levels are associated with lower ejection fraction, higher rates of hypertension and diabetes, and major adverse cardiac events (21, 24), as well as worsening renal function and in-hospital and 1-year mortality (28). These clinically meaningful findings greatly spark interest in further evaluating the role of penKid as a relevant biomarker for the detection and monitoring of kidney function in the setting of cardiac surgery.

In addition, bioactive adrenomedullin (bio-ADM) is a freely circulating peptide with a short plasma half-life affecting a multitude of biological systems (29–33). Bio-ADM secretion is influenced by various inflammatory mediators and lipopolysaccharides (31, 34), of which many are frequently released during cardiopulmonary bypass (CPB)-associated inflammation and play essential roles in the pathomechanisms of shock (1). The fact that bio-ADM secretion is both inhibited and enhanced by several of these substances suggests that the close regulation of bio-ADM is necessary for optimal endothelial function (31, 35–37). Bio-ADM exerts many functions in the human organism, including the regulation of blood pressure, endothelial barrier function, and immunoregulation, and has cardioprotective effects, which were described in greater detail in a recent review by Geven et al. (31, 38–41). Bio-ADM correlates with disease severity and mortality in patients with septic shock, dyspnea, (42), and heart failure (28, 43–46) and discriminates between patients requiring vasopressor therapy and those not in need of circulatory support in septic shock (39, 46). Elevated bio-ADM levels are associated with the extent of myocardial injury (47) and with shock refractoriness and organ dysfunction in patients with cardiogenic shock (48). In patients with acute heart failure, bio-ADM was associated with peripheral edema, 1-year mortality, rehospitalization, and length of hospital stay (28), which altogether may render bio-ADM an attractive mediator for monitoring perioperative hemodynamic alterations in patients undergoing cardiac surgery.

These findings impose the question that, if in addition to the well-established EuroSCORE II, penKid and bio-ADM can be used as promising biomarkers in the perioperative setting of cardiac surgery to predict a prolonged ICU stay due to postoperative complications, which necessitate life-sustaining therapies.

## 2. Methods

### 2.1. Study population

This prospective observational explorative study performed at the University Hospital RWTH Aachen (Aachen/Germany) was approved by the institutional review board (Ethics Committee, RWTH Aachen University, Germany), registered at clinicaltrials.gov (NCT 02488876), and performed in adherence to the Declaration of Helsinki. Patients undergoing elective cardiac surgery were enrolled consecutively between January and June 2017. Written informed consent was obtained from all enrolled patients prior to surgery. The exclusion criteria were emergency operations, pregnancy, lack of informed consent, and age < 18 years.

All patients underwent general anesthesia with catheterization, conventional open-heart surgery with the use of aortic cross-clamping, cardioplegic myocardial arrest, and CPB according to local clinical standards. After surgery, all patients were transferred to a cardiac surgery ICU and were weaned from sedation and mechanical ventilation according to our department's standards.

### 2.2. PenKid and bio-ADM measurement

Blood samples for the measurement of penKid, bio-ADM, and inflammatory markers were collected before induction of anesthesia (S1), at the end of CPB (S2), at admission to the ICU (S3), and 24 and 48 h postoperatively (S4 and S5). The ethylenediaminetetraacetic acid (EDTA) whole blood samples were centrifuged at 3,000 rpm for 10 min, and the supernatants were transferred to cryotubes for storage at −80°C until final analysis.

The assays for the measurement of plasma bio-ADM and penKid have been previously described in greater detail (18, 39, 49). Bio-ADM 1–52 and penKid 119–159 were measured in EDTA plasma samples using immunoluminometric sphingotest^®^ bio-ADM assays (SphingoTec GmbH, Hennigsdorf, Germany), as described previously (18, 39, 49). The laboratory performing the biomarker measurement was blinded to the clinical and demographic data of the patients. The 97.5^th^ percentile for penKid and bio-ADM in healthy adult subjects is 89 pmol/L (90% CI 85–118 pmol/L) and 29 pg/ml (90% CI 27–38 pg/ml), respectively. The upper normal range of penKid (89 pmol/L) is also the clinical cutoff value for the diagnosis of AKI. The clinical cutoff value for bio-ADM for patients with sepsis and septic shock is 70 pg/ml (39, 50).

### 2.3. Data collection

All other clinical and laboratory outcome parameters were collected from chart review and performed as part of the clinical routine. Laboratory evaluations were performed as indicated by the treating medical team.

At baseline, medical records and laboratory analyses as per hospital standards were used to assess comorbidities and comedications. The EuroSCORE II was calculated according to its initial description (www.euroscore.org/calc.html) (6). Chronic kidney disease (CKD) was classified using the classification of CKD according to the Kidney Disease: Improving Global Outcomes (KDIGO) 2012 Clinical Practice Guideline for the Evaluation and Management guidelines (51). According to these KDIGO guidelines, “CKD is defined as abnormalities of kidney structure or function, present for >3 months, with implications for health and CKD is classified based on cause, GFR category, and albuminuria category.” There are five GFR categories (1 = GFR ≥ 90; 2 = 60–89; 3a = 45–59; 3b = 30–44; 4 = 15–29; 5 = < 15 ml/min/1.73 m^2^) that are used routinely in our hospital and for this study (51).

Patients undergoing cardiac surgery showed a rather short ICU stay, and most patients were discharged from ICU after one night in the ICU, so a prolonged ICU stay was defined as >24 h (52).

During surgery and ICU stay, patient data and clinical information (e.g., hemodynamics, ventilator data, vital parameters, fluid treatment, drainage from chest tubes, medication, and urine output) were recorded continuously. After ICU discharge, patients' clinical and laboratory status was evaluated daily as indicated by the medical team and part of the clinical standard. Evaluation of organ dysfunction (hemodynamic support, mechanical ventilation, and RRT) and possible adverse events was performed daily using clinical data and laboratory analyses to evaluate inflammation and organ dysfunction as per hospital routine. AKI was classified using the staging system of the Acute Kidney Injury Network (AKIN) and patients were followed up during the entire ICU stay (53). Organ dysfunctions were assessed using the Sequential Organ Failure Assessment (SOFA) score according to the original description (54).

### 2.4. Statistical analysis

The aims of this explorative study were as follows:

(a) to analyze the perioperative course of penKid and bio-ADM levels,
(b) to evaluate the predictive value of penKid and bio-ADM separately and in combination with the current gold standard of risk stratification (EuroSCORE II),
(c) to describe the association of penKid with renal disease, and
(d) to describe the association of bio-ADM with hemodynamic instability.

For the descriptive part, continuous variables were described by median and interquartile ranges (IQR) and categorical variables by counts and percentages. Group differences were tested by the Kruskal–Wallis test for the continuous variables and Pearson's chi-square test for count data for the categorical variables. For all correlations, Spearman correlation coefficients were used.

Logistic regression was used to evaluate the association of penKid and bio-ADM for dichotomous endpoints. To demonstrate independence from the EuroSCORE II, the added value of each biomarker on top of the EuroSCORE II was evaluated based on the likelihood ratio chi-square test for nested models. The concordance index (C-index or area under the curve [AUC]) is given as an effective measure for univariable and multivariable models. For multivariable models, a bootstrap-corrected version of the C-index is given.

All statistical tests were two-tailed, and a two-sided *p*-value of 0.05 was considered significant. The statistical analyses were performed using R version 3.4.3 (http://www.r-project.org, library rms, Hmisc, ROCR) and Statistical Package for the Social Sciences (SPSS) version 22.0 (SPSS Inc., Chicago, Illinois, USA).

## 3. Results

### 3.1. Patients

A total of 136 patients undergoing cardiac surgery and reflecting a representative cohort of patients undergoing cardiac surgery were consecutively enrolled within the planned period. Bio-ADM and penKid were measured. Their baseline characteristics and overall clinical results are displayed in Table 1.

**Table 1:** Baseline characteristics of patients.

**Parameter**	***n***	**Median [interquartile range] or amount (%)**
**Baseline parameter**		
Age in years	136	66 [60–73.25]
Duration of the aortic cross-clamp in min	110	73 [58.25–90.5]
Duration of CPB in min	110	113 [91.25–138.75]
Sex (women)	136	36 (26.5)
Heart failure^*^	136	
NYHA 1		1 (0.7)
NYHA 2		4 (2.9)
NYHA 3		7 (5.1)
NYHA 4		2 (1.5)
EuroSCORE II	136	4.2 [2.3–7.5]
EF%	136	
>55%		88 (64.7)
35–54%		41 (30.1)
<35%		7 (5.1)
Type of surgery	136	
Valve		84 (61.8)
CABG		22 (16.2)
Combined/ other		30 (22.1)
Chronic kidney disease before surgery	136	
CKD 1		0 (0)
CKD 2		3 (2.2)
CKD 3		3 (2.2)
CKD 4		5 (3.7)
CKD 5		0 (0)
**Outcomes**		
Acute kidney injury	134	13 (9.7)
Renal replacement therapy	136	7 (5.1)
Duration of vasopressor support in h (norepinephrine)	134	18 [12–41.75]
Duration of vasopressor support in h (adrenaline)	46	18.5 [10.5–39.5]
Extracorporeal membrane oxygenation	136	2 (1.5)
Time to postoperative extubation in h	82	12.25 [8.5–15.5]
Total duration of mechanical ventilation in h	82	9.75 [7.12–14.12]
Re-Intubation	82	3 (3.7)
ARDS	82	7 (8.5)
Stroke	136	4 (2.9)
ICU length of stay in d	136	0 [0–1]
In-hospital mortality	136	9 (6.6)
30-day mortality	136	6 (4.4)
ICU length of stay < 24 h	136	28 (20.6)
Infection	136	25 (18.4)
Wound infection	136	5 (3.7)
Combined Endpoint (ICU death, mech. ventilation, re-intubation, infection or wound infection)	**136**	**28 (20.6)**

n, number of patients; BMI, Body Mass Index; CPB, cardiopulmonary bypass; EuroSCORE, preoperative European System for Cardiac Operative Risk Evaluation Score; EF%, preoperative ejection fraction; CABG, coronary artery bypass graft;

* clinical signs of heart failure as assessed by the New York Heart Association Score Class II-IV; CKD, chronic kidney disease as classified by KDIGO; AKIN, acute kidney injury as classified by Acute Kidney Injury Network; ARDS, Acute Respiratory Distress Syndrome. Bold values indicate significant results.

### 3.2. Perioperative courses of bio-ADM and penKid

**Figure 1:**
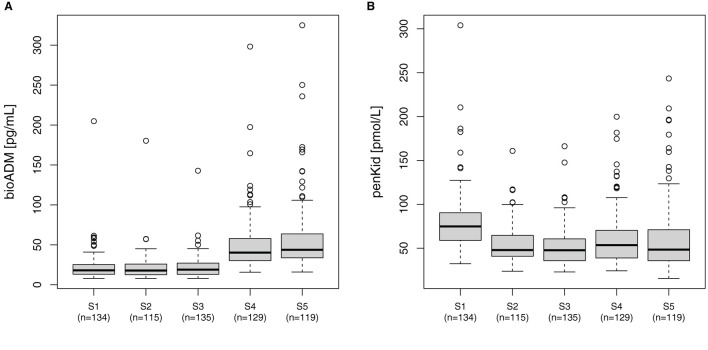
Perioperative course of **(A)** bio-ADM and **(B)** penKid. n, number of patients; S1, prior to surgery; S2, intraoperatively at the end of cardiopulmonary bypass; S3, at ICU-admission; S4, 24 h after surgery; and S5, 48 h after surgery; bio-ADM, bioactive Adrenomedullin; penKid, proenkephalin A.

In all patients, the median bio-ADM levels increased over time from 18 pg/ml [IQR 13–25] before surgery (S1) to 44 pg/ml [IQR 34–65] at 48 h after surgery (S5). Correspondingly, the perioperative levels of penKid decreased at the same time points from preoperative (S1) 75 pmol/L [IQR 59–90] to 48 pmol/L [IQR 41–65] after removal of the aortic cross-clamp (S2) and remained low, as shown in Figure 1.

### 3.3. PenKid and bio-ADM to predict a prolonged ICU-stay

While bio-ADM levels were only lightly elevated 24 h after surgery (S4) in those patients with prolonged ICU stay, the penKid levels were capable of discriminating these patients more distinctly at all time points. Regarding the prediction of a prolonged and thus possibly complicated ICU stay, we calculated the predictive accuracy for prolonged ICU stay for both biomarkers. The addition of penKid and bio-ADM to the EuroSCORE II for predicting an ICU LOS of >24 h was statistically significant after the removal of the aortic cross-clamp (S2) and 24 h after surgery (S4) for penKid and bio-ADM. Bio-ADM did not provide additional value to penKid. The largest AUC was obtained when combining EuroSCORE II with penKid measured 24 h after surgery with a significant additional value (*p* < 0.05).

**Figure 2:**
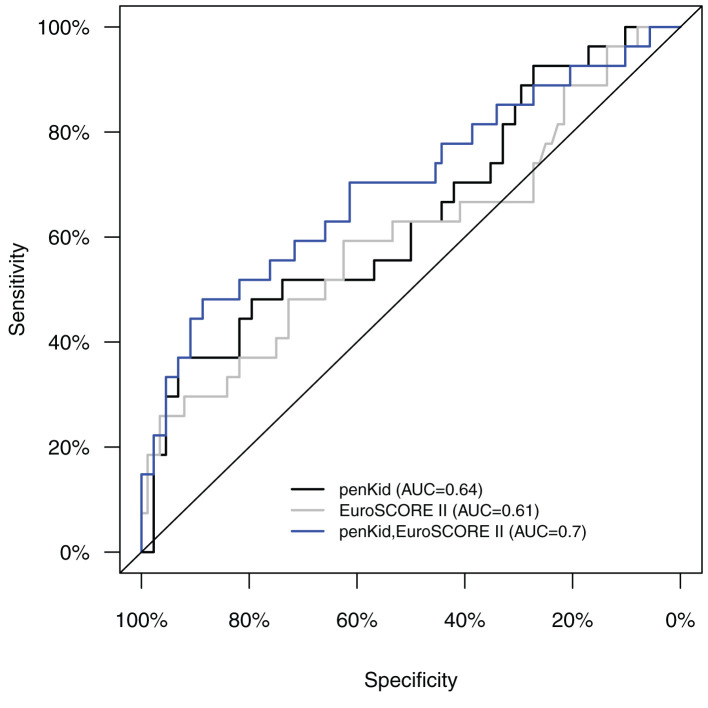
AUC/ROC analysis of intraoperative levels of penKid and EuroSCORE II alone (S2) and a combination of penKid (S2) with preoperative EuroSCORE II for the combined outcome “ICU length of stay >1 day or in-hospital death.” AUC/ROC, area under the curve/receiver operating characteristics; penKid, proenkephalin A; S2, intraoperatively at the end of cardiopulmonary bypass; EuroSCORE II, preoperative European System for Cardiac Operative Risk Evaluation Score II.

Serial measurements of penKid and bio-ADM revealed a statistically significant value of the addition of penKid to EuroSCORE II for the time points after removal of the aortic cross-clamp (start of reperfusion) (S2), at ICU admission (S3), and 24 h after surgery (S4), as shown in Table 2 and Figure 2. The addition of bio-ADM to EuroSCORE II did not add a predictive value to EuroSCORE II.

**Table 2:** AUC analysis of serial penKid and bio-ADM and the combined outcome “ICU length of stay >1 day or death in hospital”.

	**ICU length of stay** >**1 day or in-hospital death**				
	**Pre-operatively (S1)**	**After CPB (S2)**	**ICU-admission (S3)**	**Post-operatively (24 h, S4)**	**Post-operatively (48 h, S5)**
Patients/events (*n*)	134/31	115/27	135/31	129/30	120/26
bio-ADM	0.456	0.484	0.614	0.670	0.593
penKid	0.593	**0.643** ^#^, ^*^	**0.640** ^#^, ^*^	**0.685** ^#^, ^*^	**0.640** ^#^
EuroSCORE II	**0.594** ^#^	**0.606** ^#^	**0.602** ^#^	**0.612** ^#^	**0.589** ^#^

# *p* < 0.05,

* added value to EuroSCORE II. AUC, area under the curve/receiver operating characteristics; bio-ADM, bioactive Adrenomedullin; CPB, cardiopulmonary bypass; ICU, intensive care unit; penKid, proenkephalin A; EuroSCORE II, preoperative European System for Cardiac Operative Risk Evaluation Score II. Bold values indicate significant results.

### 3.4. Association of penKid with renal disease

PenKid was significantly associated with preexisting CKD (*n* = 11) (median 71.4 pmol/L [IQR 58.3–87.0] in patients without CKD vs. median 105.7 pmol/L [IQR 91.4–172.3] in patients with CKD, *p* = 0.0001). The observed difference remained present for all measurement time points in the postoperative period (all p < 0.01, refer also to Supplementary Figure S1A). Elevated penKid levels were associated with the severity of CKD (p < 0.02 for each time point) as displayed in Supplementary Figure 1B with patients with higher CKD stages having higher penKid levels.

**Figure 3:**
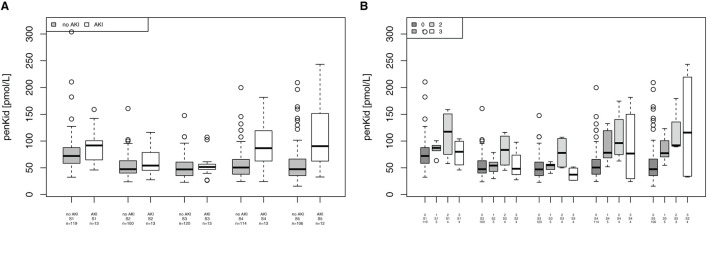
Association of proenkephalin A (penKid) with acute kidney injury (AKI). **(A)** Comparison of penKid levels in patients with/without AKI. **(B)** penKid levels in patients by Acute Kidney Injury Network (AKIN) class. n, number of patients; S1, prior to surgery; S2, intraoperatively at the end of cardiopulmonary bypass; S3, at ICU admission; S4, 24 h after surgery; and S5, 48 h after surgery.

Mean blood levels of penKid were significantly elevated in patients undergoing cardiac surgery at 24 h (S4) in patients without AKI vs. patients with AKI (median 50.5 pmol/L [IQR 37.9–65.6] vs. median 86.6 pmol/L [IQR 62.7–119.3], *p* = 0.0002) and at 48 h (S5) in patients without AKI vs. patients with AKI (median 47.3 [IQR 35.4–66.0] vs. median 90.4 pmol/L [IQR 66.4–137.3; *p* = 0.0027), as shown in Figure 3A. PenKid levels were associated with the severity of AKI at 24 h (S4) and 48 h (S5) after surgery (*p* = 0.009 and 0.0125, respectively), as displayed in Figure 3B.

Furthermore, there was a tendency that elevated penKid levels were associated with the need for RRT, as shown in Appendix S2. This was observable preoperatively (*p* = 0.079 at S1) and immediately after the removal of the aortic cross-clamp (p = 0.063 at S2), while toward later measurement points, the difference was smaller [*p* = 0.500 at ICU admission (S3), *p* = 0.137 after 24 h (S4), and p = 0.402 after 48 h (S5)]. Supplementary Figure 2 provides an overview of the association between penKid with RRT. Supplementary Figure 3 provides an association between penKid with AKI or with or without CKD.

### 3.5. Association of bio-ADM with markers of shock

While bio-ADM was correlated with lactate immediately post-surgery (*r* = 0.39, *p* < 0.001 at ICU admission), it did not correlate with lactate at 24 h or 48 h after surgery (*r* = 0.15, *p* = 0.098 at 24 h and r = 0.03, *p* = 0.781 at 48 h). Bio-ADM was also correlated with the cumulative noradrenaline dosage: at ICU admission (*r* = 0.20, *p* = 0.024), 24 h after surgery (*r* = 0.28, *p* = 0.001), and 48 h after surgery (*r* = 0.29, *p* = 0.001).

### 3.6. Association of penKid and bio-ADM with non-renal complications

**Figure 4:**
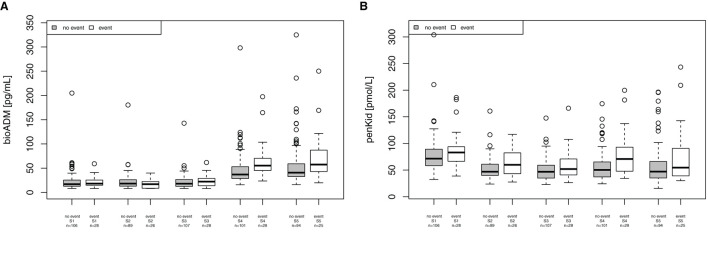
Association of bioactive Adrenomedullin (bio-ADM) and proenkephalin A (penKid) with the combined endpoint “in-hospital mortality, patients receiving mechanical ventilation support, re-intubation, post-surgery infection and wound infections” **(A)** bio-ADM and **(B)** penKid; n, number of patients; S1, prior to surgery; S2, intraoperatively at the end of cardiopulmonary bypass; S3, at ICU admission; S4, 24 h after surgery; and S5, 48 h after surgery.

For the combined endpoint of “in-hospital mortality, patients receiving mechanical ventilation support, re-intubation, post-surgery infection and wound infections,” bio-ADM was elevated at 24 h and 48 h after surgery, as shown in Figure 4A (24 h (S4): 37 [28.7–53.0] pg/ml in patients without complications and 55.2 [45.6–69.2] pg/ml in patients with complications, *p* < 0.001, AUC 0.709; 48 h (S5): 40.7 [32.8–59.0] pg/ml and 57.4 [43.0–87.2] pg/ml, respectively, *p* = 0.020, AUC 0.651). The EuroSCORE II did not reach statistical significance for this endpoint (*p* = 0.154). PenKid was only significantly elevated 24 h (S4) after surgery (50.2 [37.1–65.2] pmol/L in patients without complications and 70.8 [49.8–92.8] pmol/L in patients with complications, p = 0.002, AUC 0.692), as shown in Figure 4B.

## 4. Discussion

The present data from an explorative observational study that identified the circulating levels of penKid decreased over time. PenKid was a useful marker to predict a prolonged ICU LOS, which added prognostic value to the risk prediction of the well-established EuroSCORE II, when measured after the end of CPB and 24 h after cardiac surgery. PenKid also added prognostic value to the EuroSCORE II for the combined outcome “ICU LOS >1 day and in-hospital mortality,” while bio-ADM did not. PenKid was associated with CKD and the incidence of AKI and RRT, as well as with the severity of AKI. Bio-ADM measured 24 h after surgery was useful to predict a prolonged ICU-LOS as well. Bio-ADM was correlated with lactate immediately post-surgery and with the cumulative noradrenaline dosage.

Our results regarding penKid are supported by a study by Mossanen et al. (22), who observed significantly higher preoperative penKid levels in patients developing AKI than in patients without AKI in a cohort of 107 patients undergoing elective cardiac surgery. When excluding patients with preexisting CKD, a known predictor for postoperative renal complications, the penKid differences became insignificant (22). Similar to their results, we also observed a decrease in penKid levels from preoperatively to intraoperatively and postoperatively. A potential explanation is a connection with volume status, drugs, and reduced production of penKid during cardiac arrest, tissue hypoperfusion, and perhaps hypothermia, since many non-neuronal tissues (e.g., heart, skeletal muscle, intestines, and kidney) produce penKid (19). In 2015, Shah et al. reported a significant correlation between elevated preoperative and postoperative penKid levels and AKI incidence after cardiac surgery in a cohort of 92 patients. The AUC/ROC was 0.683 for penKid and 0.721 for creatinine measured preoperatively (23). Changes in the penKid levels, from preoperatively to 12 h postoperatively, had the greatest AUC/ROC for AKI after postoperative day 1.

Our study confirms the findings from Mossanen et al. (22) and Shah et al. (23). In addition, we identified a potential prognostic relevance through a combination of penKid with the well-established EuroSCORE II.

### 4.1. Prognostic scoring systems and biomarkers in cardiac surgery

The ICU LOS of patients undergoing elective cardiac surgery is usually short, and the majority of patients spends only the first postoperative night in the cardiac ICU and is discharged to intermediate care or standard care on the first postoperative day. However, patients with complex surgical procedures and more comorbidities, as well as patients who are malnourished and frail, have an increased risk for longer ICU and hospital LOS as well as mortality (3, 55, 56). A variety of risk prediction models have been developed and validated in the past (57). The EuroSCORE was developed in 1999 and had an AUC/ROC of 0.76 in the initial validation dataset (5) and an AUC/ROC of 0.75–0.78 in over 400,000 patients undergoing cardiac surgery in North America (58). The updated EuroSCORE II was proposed in 2012 with an AUC/ROC of 0.81 (6). The STS risk model is widely accepted as the gold standard in the United States and has been updated in 2018. The C-statistics vary regarding the endpoints (composite outcome morbidity and mortality of 0.71–0.74 depending on the population) (7, 8). EuroSCORE II and STS score performed similarly in one meta-analysis (9). In another meta-analysis, the EuroSCORE II, EuroSCORE I, and STS risk score had AUC/ROCs of 0.844, 0.819, and 0.846 (10), respectively. Nevertheless, the European Association for Cardio-Thoracic Surgery (EACTS) states that the current risk models have been shown to be poorly calibrated, particularly in high-risk patients, indicating a need for quality improvement in risk prediction (11).

Therefore, the addition of biomarkers to these models hypothetically might improve the predictive values of these clinical scores. A previous attempt to add a panel of biomarkers (ST2, galectin-3, n-terminal pro-brain natriuretic peptide, cystatin C, and interleukins 6 and 10) to an established risk model (STS) has been shown to improve the AUC/ROC from 0.66 to 0.74 (*p* < 0.0001) in the derivation cohort, but external validation was poor (AUC/ROC 0.51) (16). Lurati Buse et al. (17) added troponin T (TNT) and brain natriuretic peptide (BNP) to the original EuroSCORE and observed a better prediction of 1-year all-cause mortality and the occurrence of major adverse cardiovascular events. The net reclassification index of the addition of TNT and BNP to the EuroSCORE was 0.276 (95% confidence interval, 0.195–0.348), allowing for improved risk stratification in every fourth patient. Attempts are currently made to further improve the EuroSCORE by including social, functional, emotional, and behavioral factors (59).

Regarding the two discussed biomarkers, in 2022, van Lier et al. (60) described a significant association between bio-ADM with prolonged vasopressor dependency (AUC/ROC=0.82), with AKI (AUC/ROC = 0.87) and prolonged ICU stay (AUC/ROC = 0.82) in 203 patients undergoing cardiac surgery, which could not be confirmed by our study results. In 2020, Gombert et al. found a significant correlation between penKid and AKI within 48 h after surgery at 12 h (AUC/ROC of 0.752, *p* = 0.004) and at 48 h (AUC/ROC of 0.714, *p* = 0.021) after admission to the ICU in a cohort of 33 patients undergoing elective open or endovascular thoracoabdominal aortic repair (61).

In our study, the AUC/ROC of the EuroSCORE II regarding the outcome was significantly improved through the addition of penKid measured intraoperatively or at 24 h after surgery regarding the outcomes “ICU LOS >24 h” and “ICU-LOS >24 h plus mortality.” It must be pointed out that, in our observational study, the EuroSCORE II had a relatively low AUC/ROC of between 0.594 and 0.643 compared to the abovementioned literature, which is likely due to the small sample size of 136 patients undergoing elective cardiac surgery.

### 4.2. Strengths and limitations

The strengths of our study include (a) the consecutive inclusion of an unselected broad population undergoing various cardiac surgeries with the use of CPB, (b) the standardized adjudication of adverse events by non-research personnel (experienced intensive care physicians and cardiothoracic surgeons), (c) the external measurement of biomarkers by personnel unaware of the patient's clinical course, and (d) the focus on preoperative, intraoperative, and postoperative values of the biomarkers.

We are aware of some limitations of our study: The small sample size leads to limited generalizability and our results can only be regarded as hypothesis-generating. The sample size does not allow for drawing any confirmatory conclusions regarding high-risk patients due to the complexity of surgery and comorbidities or to allow for subgroup analysis regarding the different types of surgeries. The overall good outcome of these unselected patients with low mortality, short ICU and hospital LOS, and a low rate of adverse events, such as AKI, cardiovascular events, and pulmonary complications, prohibited further subgroup analysis. In addition, we observed overall lower AUC/ROC values for EuroSCORE II, bio-ADM, and penKid, compared to previous studies, which might be due to the small sample size and the overall relatively healthy study population, leading to low mortality rates as well as short ICU LOS. Finally, we evaluated the addition of the two biomarkers to EuroSCORE II as prognostic parameters but did not do so for the STS model since it is not used in clinical routine in our hospital.

## 5. Conclusion

In the present study, the biomarkers penKid and bio-ADM were correlated with clinically significant patient outcomes after cardiac surgery, such as markers of kidney failure and shock. The findings highlighted the potential role of penKid as a useful marker to predict a prolonged ICU LOS. Furthermore, penKid added a significant prognostic value to the risk prediction of the well-established EuroSCORE II and was capable of predicting mortality.

## Data availability statement

The original contributions presented in the study are included in the article/Supplementary material, further inquiries can be directed to the corresponding authors.

## Ethics statement

The studies involving human participants were reviewed and approved by Ethics Committee, RWTH Aachen University, Germany. The patients/participants provided their written informed consent to participate in this study.

## Author contributions

AH and CS equally contributed to the conception and design of the research together with JS and DB. AH and CS drafted the manuscript together with DB, T-PS, and JS. AH, DB, T-PS, and RZ contributed to the acquisition of data. JS and GM assisted in conceiving the study and revising the manuscript. All authors contributed to the analysis and interpretation of the reviewed data, critically revised the manuscript, agree to be fully accountable for ensuring the integrity and accuracy of the work, and read and approved the final manuscript.
