# Supplementary material for: Proenkephalin A and bioactive adrenomedullin are useful for risk prognostication in cardiac surgery
Source: Front Cardiovasc Med. 2023 Jan 23;9:1017867. doi: 10.3389/fcvm.2022.1017867 (PMC9900105; doi:10.3389/fcvm.2022.1017867)
Supplement: Supplementary file 1 [file Data_Sheet_1.pdf]

frontiers

Proenkephalin A and bioactive Adrenomedullin are useful for Risk Prognostication in Cardiac Surgery

Supplementary Material

Aileen Hill[1,2]*, Deborah Bergmann[3], Janin Schulte[3], Rashad Zayat[4], Gernot Marx[1], Tim-Philipp Simon[1,] Jana Mossanen[1], Anne Brücken[1] and Christian Stoppe[5]*

[1]Department of Intensive Care and Intermediate Care, Medical Faculty RWTH Aachen, Aachen, Germany

[2]Department of Anesthesiology, Medical Faculty RWTH Aachen, Aachen, Germany

[3]SphingoTec GmbH, D-16761 Hennigsdorf, Germany

[4]Department of Cardiothoracic Surgery, Medical Faculty RWTH Aachen, Aachen, Germany

[5]Department of Anesthesiology, Intensive Care, Emergency and Pain Medicine, University Hospital Wuerzburg, Wuerzburg, Germany

*** Correspondence:**

Aileen Hill	Christian Stoppe
ahill@ukaachen.de	Christian.stoppe@gmail.com

1 Supplementary Figures

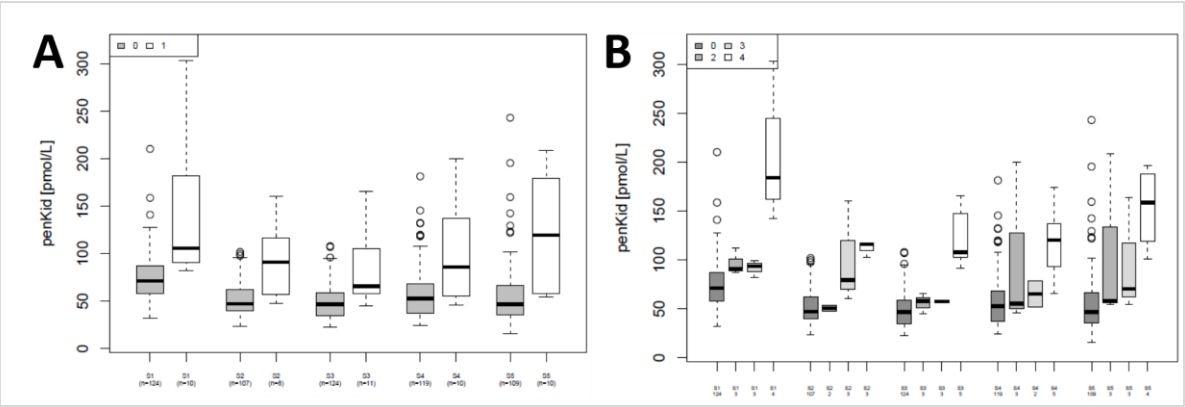

Figure S1: Correlation of penKid and chronic kidney disease (CKD), A: Comparison of penKid levels in patients with or without CKD; B: penKid levels in patients with CKD stratified by stage according to KDIGO (Kidney Disease: Improving Global Outcomes), n= number of patients, S1= prior to surgery; S2= intraoperatively at the end of cardiopulmonary bypass; S3= at ICU-admission; S4= 24 hours after surgery; and S5= 48 hours after surgery, penKid= proenkephalin

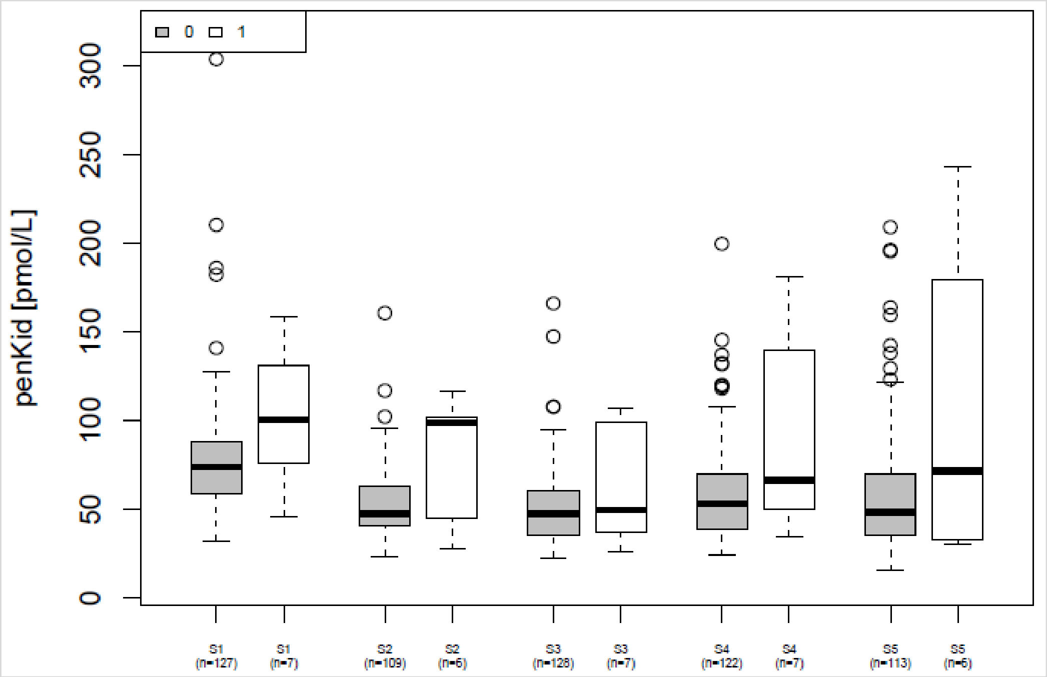

Figure S2: penKid and incidence of renal replacement therapy (RRT), penKid= proenkephalin

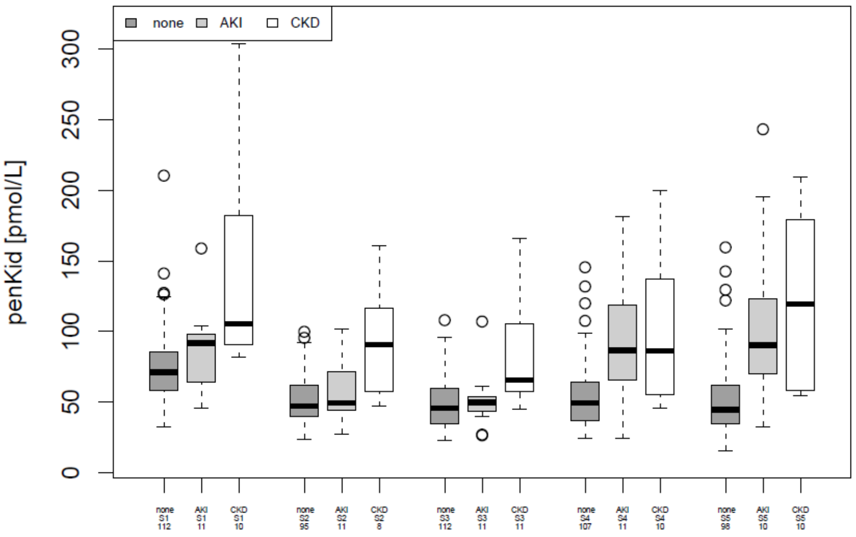

Figure S3: Association of penKid with no kidney injury (none), with acute kidney injury (AKI), but not CKD, and CKD. n= number of patients, S1= prior to surgery; S2= intraoperatively at the end of cardiopulmonary bypass; S3= at ICU admission, S4= 24 hours after surgery, and S5= 48 hours after surgery, penKid= proenkephalin, AKI= acute kidney injury, CKD= chronic kidney disease
